# Associations between Familial Rates of Psychiatric Disorders and De Novo Genetic Mutations in Autism

**DOI:** 10.1155/2017/9371964

**Published:** 2017-11-08

**Authors:** Kyleen Luhrs, Tracey Ward, Caitlin M. Hudac, Jennifer Gerdts, Holly A. F. Stessman, Evan E. Eichler, Raphael A. Bernier

**Affiliations:** ^1^University of Washington School of Medicine, Seattle, WA 91895, USA; ^2^School of Psychology, Family, and Community, Seattle Pacific University, Seattle, WA 98119, USA; ^3^Department of Psychiatry and Behavioral Sciences, University of Washington, Seattle, WA 91895, USA; ^4^Department of Genome Sciences, University of Washington, Seattle, WA 91895, USA; ^5^Department of Pharmacology, Creighton University Medical School, Omaha, NE 68178, USA; ^6^Howard Hughes Medical Institute, Seattle, WA 91895, USA

## Abstract

The purpose of this study was to examine the confluence of genetic and familial risk factors in children with Autism Spectrum Disorder (ASD) with distinct de novo genetic events. We hypothesized that gene-disrupting mutations would be associated with reduced rates of familial psychiatric disorders relative to structural mutations. Participants included families of children with ASD in four groups: de novo duplication copy number variations (DUP, *n* = 62), de novo deletion copy number variations (DEL, *n* = 74), de novo likely gene-disrupting mutations (LGDM, *n* = 267), and children without a known genetic etiology (NON, *n* = 2111). Familial rates of psychiatric disorders were calculated from semistructured interviews. Results indicated overall increased rates of psychiatric disorders in DUP families compared to DEL and LGDM families, specific to paternal psychiatric histories, and particularly evident for depressive disorders. Higher rates of depressive disorders in maternal psychiatric histories were observed overall compared to paternal histories and higher rates of anxiety disorders were observed in paternal histories for LGDM families compared to DUP families. These findings support the notion of an additive contribution of genetic etiology and familial factors are associated with ASD risk and highlight critical need for continued work targeting these relationships.

## 1. Introduction

Autism Spectrum Disorder (ASD) is a neurodevelopmental disorder characterized by impairments in social communication and repetitive and restricted interests or behaviors [1]. The rate of diagnosis is estimated to affect as many as 2% of individuals in the US [2]. The etiology of ASD is likely a combination of genetic and environmental factors [2, 3]. Heritability of ASD is estimated to be as high as 90%, suggesting that genetics contribute significantly to ASD biology [4]. While no single event accounts for more than 1% of all cases of ASD, recent studies have identified putative causal genetic events in the form of de novo copy number variations (CNV) and likely gene-disrupting mutations (LGDM). Several hundred mutations have already been identified with estimates suggesting up to 1,000 ASD risk genes ([5], Sanders et al., 2012). As such, de novo mutations may account for 30% of all cases and up to 45% of female cases of ASD [5–7]. Recent evidence suggests increased maternal transmission rate of deleterious mutations and de novo CNV deletions that more often occur on the maternal haplotype [8]. However, exome studies indicate that de novo LGDMs are associated with the paternal line [9] and the transmission rate of ASD-associated paternal de novo point mutations increases with paternal age [5].

Elevated rates of psychiatric disorders have been consistently reported among the relatives of individuals with ASD, including schizophrenia, anxiety, depression, obsessive compulsive disorder, and bipolar disorder [10–18]. Long-standing evidence suggests that psychiatric disorders aggregate in family members affected by ASD [15, 16, 19, 20]. In addition, several studies indicate that the onset of familial psychiatric diagnoses originates before the proband's diagnosis, suggesting that vulnerability associated with psychiatric disorders may occur independently of psychosocial stressors related to the proband [11, 13]. Family members of individuals with ASD also exhibit elevated incidences of ASD-related traits known as the broader autism phenotype, underscoring the dimensional familial nature of ASD-related symptoms [15, 17, 21]. Maternal and paternal psychiatric histories vary considerably from an epidemiological standpoint as well as transmission rates of maternal and paternal de novo mutations [8, 9, 13], necessitating the separate examination of maternal and paternal factors.

Findings increasingly support the presence of common molecular and genetic features between ASD and other psychiatric disorders [22–29]. Shared genetic contributions between psychiatric disorders and ASD etiology indicate that unstable or deleterious underlying genetic architecture may lead to increased genetic vulnerability in ASD [11, 15, 16, 30]. For instance, recent work suggests that family history of psychiatric illness and de novo genetic events influence phenotypic ASD profiles [31].

Despite the confluence of genetic (e.g., de novo mutations) and familial (e.g., history of psychiatric illness) factors as major contributors to ASD risk, the extent by which these factors interact is less clear. Considering that familial psychiatric factors likely involve both genetic and environmental mechanisms, it is possible that these factors impact children with distinct genetic events in different ways. For instance, Girirajan and colleagues [32] proposed the multiple or “second hit” hypothesis for ASD, such that, in addition to a primary genetic event, a secondary insult is necessary to result in a more severe clinical presentation of ASD. This second hit could potentially be another unidentified ASD-associated genetic event or an environmental factor that influences development and confers ASD risk.

Here, in this exploratory study, we sought to test whether the presence of a psychiatric disorder in family members acts as a potential “second hit.” Specifically, we targeted the potential additive risk for ASD from de novo genetic events and elevated familial psychiatric burden. We focused on families with a child with ASD and either a CNV deletion, CNV duplication, or LGDM who had completed a detailed interview regarding maternal and paternal family history of psychiatric illness. Building upon the “second hit” hypothesis for ASD, we hypothesized that less severe “first hit” mutations (CNV deletion or duplication) would be associated with higher rates of familial psychiatric disorders than more severe “first hit” mutations (truncating LGDM). In this way, we can evaluate whether an additional “second hit” insult (i.e., elevated familial psychiatric burden) is present with the less severe CNV duplication phenotype.

## 2. Materials and Methods

### 2.1. Participants

Participants (*N* = 2514) included families of 4- to 18-year-old children with ASD who had previously participated in genetic testing as part of the Simons Simplex Collection (SSC) with valid and complete medical history data. The objective of the SSC was to identify de novo genetic variants that contribute to the overall risk of ASD by focusing on simplex families (families with one child with ASD) without a family history of ASD [33]. Each family (i.e., biological parents, proband with ASD, and unaffected siblings) provided DNA via blood sample for sequencing and completed rigorous clinical and behavioral characterization of the proband. For all groups, ASD diagnosis was confirmed as part of inclusion criteria for the study using the Autism Diagnostic Interview-Revised [34], Autism Diagnostic Observation Schedule [35], and expert clinical judgment (APA, 2000). Families with complete family mental health history information were divided into groups based upon whether their child had an identified ASD-associated de novo genetic event as identified in [36]. The three genetic etiology groups included children with (1) a likely gene-disrupting mutation (LGDM group, *n* = 267, without any other relevant CNVs), (2) recurrent or rare CNV deletion event (DEL group, *n* = 74), and (3) recurrent or rare CNV duplication event (DUP group, *n* = 62). The remaining families whose child did not have an ASD-associated genetic event were included as a control group (NON group, *n* = 2111). Participant characteristics are provided in Table 1. The three supplementary tables in Supplementary Material available online at https://doi.org/10.1155/2017/9371964 list the participants' specific genetic events as identified by Sanders et al., 2015 (S1, DEL events; S2, DUP events; and S3, LGDM events). A series of one-way ANOVAs indicated no significant differences in age, verbal IQ, internalizing problems, or externalizing problems (Tukey HSD correction, all *p* > .095). There was a significant group difference of nonverbal IQ (*F*(3, 2312) = 7.75, *p* < .01) related to reduced nonverbal IQ for the DEL and LGDM groups relative to the NON group (*p*'s < .025). The investigation was carried out in accordance with the most recent version of the Declaration of Helsinki and reviewed by the institutional review board. Written informed consent was obtained from participants including parents and children. Assent was obtained if developmental age was above 7 years in accordance with the ethical standards approved by the local institutional review board.

### 2.2. Familial Rate of Psychiatric Disorders Measure

Information regarding family history of psychiatric disorders was collected through semistructured caregiver interviews conducted as part of the SSC [33]. Caregivers identified the number of individuals that had been diagnosed with psychiatric disorders separately for maternal and paternal histories. Possible individuals included the proband's aunts, uncles, and grandparents. Due to exclusionary criteria, immediate family members with a history of schizophrenia were not eligible to participate in SSC or subsequently in this analysis. The following eight categories were created based on the type of psychiatric disorder endorsed. Diagnoses were categorized according to the Diagnostic and Statistical Manual of Mental Disorders, 5th edition [1]: (1) depressive disorders (depression disorder, dysthymic disorder, and other psychological disorders), (2) anxiety disorders (anxiety disorder and social phobia), (3) obsessive compulsive disorder, (4) trauma and stress (adjustment disorder and posttraumatic stress disorder), (5) neurodevelopmental disorders (ADHD, behavior disorder, and Tourette's syndrome), (6) bipolar disorders, (7) feeding and eating disorders (eating disorder and pica), and (8) schizophrenia (schizophrenia and other psychotic disorders). Personality disorders were initially included; however, no indices of personality disorders were reported and, subsequently, this category was removed from analysis. Given the exclusion criteria for participation in the Simons Simplex Collection, none of the immediate or extended family members had been diagnosed with ASD.

To determine rates of psychiatric disorders, every family member that the respondent endorsed for a particular diagnostic category was given a value of 1. The overall sum was totaled for each participant separately for maternal and paternal family histories. To avoid false negatives related to the age-dependent nature of diagnosing many psychiatric disorders, siblings, half-siblings, and cousins were not included. The total score was then divided by the number of family members evaluated to produce a ratio that could be compared among families of different sizes, labeled as rate of psychiatric disorders separately for maternal and paternal rates. Only participants with complete quantitative family information were included.

### 2.3. Statistical Analysis

Rates of psychiatric disorder were analyzed using mixed modeling in SAS software version 9.4 (SAS Institute, Inc., Cary, NC, USA). The full-factorial model consisted of fixed effects and all possible interactions for kind of psychiatric disorder (8: depressive, anxiety, obsessive compulsive disorder, trauma/stress, neurodevelopmental, bipolar, feeding/eating, and schizophrenia), familial relation (2: maternal and paternal), and group (4: DEL, DUP, LGDM, and NON). The model was estimated using maximum likelihood, and fixed effects were tested by *F*-tests with Satterthwaite approximation of denominator degrees of freedom. Our primary hypothesis was to test group differences and potential familial relation interactions as main effects and also to consider secondary group differences related to specific kinds of psychiatric disorders. Thus, a priori pairwise comparisons were generated using least square means with Tukey correction for multiple comparisons [37].

## 3. Results

**Figure 1:**
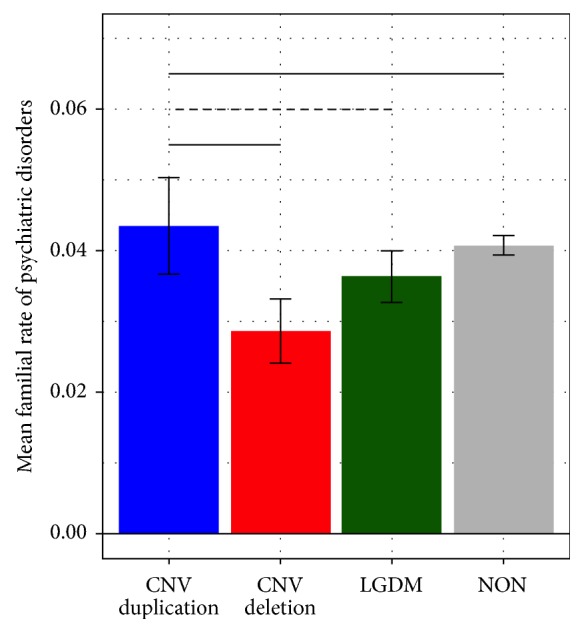
Group differences in familial rate of psychiatric disorders. Mean rate of psychiatric disorders is reported with standard error. Horizontal lines represent significance (solid line, *p* < .05; dashed line, *p* < .1).

**Figure 2:**
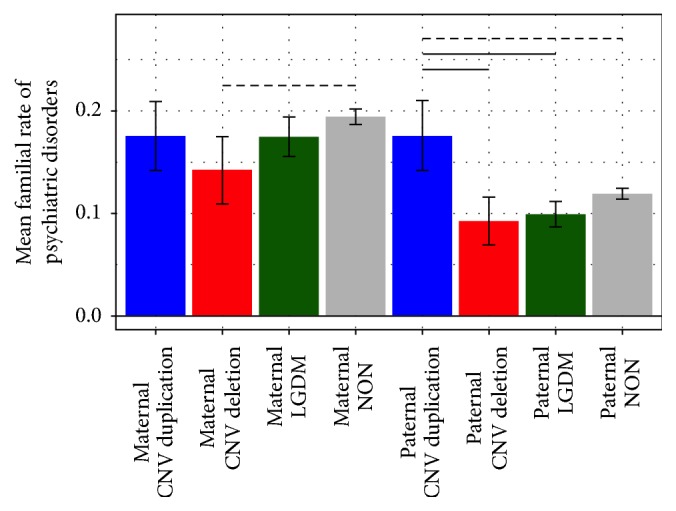
Group differences in familial rate of psychiatric disorders by familial relation. Mean rate of psychiatric disorders is reported with standard error. Horizontal lines represent significance (solid line, *p* < .05; dashed line, *p* < .1).

Mixed model fixed effects results are presented in Table 2, and least square means group comparisons are presented in Table 3. Results indicated an omnibus effect of genetic group (*F*(3, 40000) = 2.70,* p* = .044) such that DUP families exhibited increased rates of psychiatric disorders compared to DEL (*t*(6400) = 2.21,* p *= .027) and NON (*t*(40000) = 2.01,* p* = .044) and marginally significantly increased rates compared to LGDM (*t*(40000) = 1.65,* p *= .098) (Figure 1). This pattern of genetic group differences was evident for paternal rate [DEL < DUP,* t*(40000) = 2.06,* p *= .039; LGDM < DUP,* t*(40000) = 2.32,* p *= .21; and NON < DUP,* t*(40000) = 1.87,* p* = .062]. In contrast, there was only a marginal effect of maternal rate, such that DEL families had reduced rates of psychiatric disorders compared to NON families (*t*(40000) = 1.88,* p* = .060) (Figure 2). Overall, maternal rate was increased compared to paternal rate (*F*(1, 40000) = 10.70,* p* = .001), particularly within the LGDM (*t*(40000) = 3.72,* p* = .0002) and NON (*t*(40000) = 10.39,* p* < .0001) groups.

**Figure 3:**
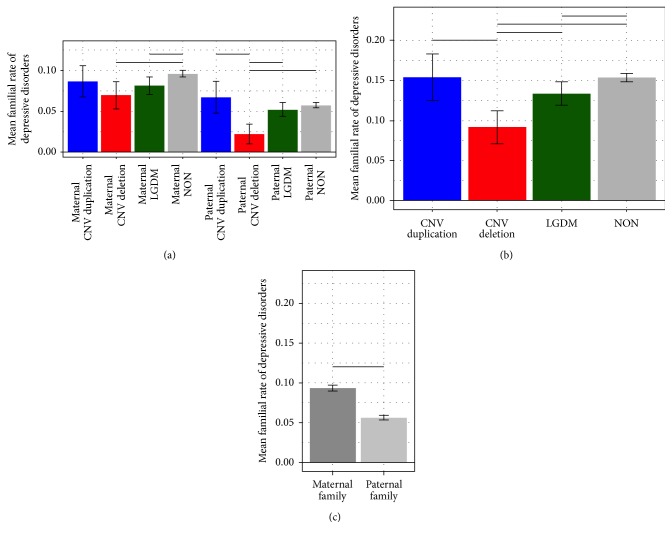
Familial rate of depression. Mean rate of depressive disorders is reported with standard error. Horizontal lines represent significance (*p* < .05). (a) Interaction between group and familial relation. (b) Main effect of group. (c) Main effect of familial relation.

Genetic group differences were the most prominent for depressive disorders overall with the DEL group exhibiting reduced rates of psychiatric disorders compared to DUP, LGDM, and NON groups (*t'*s > 2.73,* p*'s < .006) (Figure 3(b)). Closer inspection indicated that this pattern was evident for paternal rates (*t'*s > 2.76,* p*'s < .006), but the DEL group was only reduced relative to the NON group for maternal rates (*t*(40000) = 2.72,* p* = .007) (see Figure 3(a)). The LGDM group exhibited reduced rates of depressive disorders relative to the NON group for maternal rates (*t*(40000) = 2.75,* p* = .006) but not paternal rates (*t*(40000) = .96,* p* = .34), driving an overall effect between LGDM and NON (*t*(40000) = 2.62,* p* = .009) (Figure 3(b)). Of note, the rate of depressive disorders was greater overall for maternal compared to paternal families (*t* = 4.84,* p* < .0001) (Figure 3(c)). Lastly, LGDM families had a reduced rate of paternal anxiety disorders compared to DUP families [paternal,* t* = 2.06,* p* = .04; maternal,* t* = 0.34,* p* = .74]. There were no group differences in rates of obsessive compulsive disorder, trauma and stress, neurodevelopmental disorders, bipolar disorders, feeding and eating disorders, or schizophrenia.

## 4. Discussion

Our aim was to assess the familial rates of psychiatric disorders in relation to likely pathogenic genetic factors identified in children with ASD. We found that children with de novo CNV duplications have a significantly greater familial incidence of psychiatric disorders than children with de novo deletions and show a similar trend with likely gene-disrupting mutations. Critically, this increased rate in families with children with CNV duplication is similar to the rate of a control group of children without an ASD-associated genetic event. We also examined the contribution of psychiatric burden from maternal and paternal family histories separately and across genetic groups. Our results indicate that paternal rate of psychiatric disorders was greater in duplications than in deletions and LGDMs.

These results suggest that family psychiatric history may play a differential role in ASD depending on the type of genetic event contributing to ASD for a given individual. One explanation for the increased rate of psychiatric disorders for family members of children with duplications could be that duplications confer an overall psychiatric risk that necessitates an additional genetic disruption to result in a psychiatric presentation such as ASD [32]. In support of the “second hit” hypothesis, our results suggest that psychiatric burden (second hit) and the presence of a de novo CNV duplication (first hit) interrelate in a multiplicative manner, resulting in the development of the ASD phenotype. In other words, compared to deletion CNVs, duplication CNVs potentially may inflict a less severe impact on the ASD phenotype; however, the presence of both hits (CNV duplication and familial psychiatric burden) leads to the development of ASD. This “second hit” hypothesis is further strengthened by the fact that the de novo duplication group exhibited increased rates of familial psychiatric disorders to the idiopathic (NON) families whose children have no known genetic etiology.

While Girirajan and colleagues [32] show an inverse relationship between de novo cases and the prevalence of a “second hit,” we limited our sample to only de novo CNV duplications/deletions and LGDMs without any additional CNVs. Therefore, our results extend the “second hit hypothesis” by proposing that individuals with de novo duplications have higher penetrance (relative to other de novo cases) due to a family history of psychiatric burden. As such, perhaps the presence of CNVs and LGDM confers independent risk factors for ASD and the “second hit” (psychiatric burden) uniquely exacerbates the ASD phenotype.

An alternative hypothesis is that family psychiatric history provides a vulnerable background upon which a CNV's deleterious effect can more easily be observed or propels an individual across a diagnostic threshold, such that the family psychiatric status can serve as either a protective or risk factor. This hypothesis is supported by evidence that individuals with a* 16p11.2 *locus CNV (i.e., duplication or deletion) show quantitative decrements in phenotypic domains that are reflective of the parental phenotype [38]. According to this perspective, a deleterious LGDM effect would prove more impactful such that family psychiatric history is less of a protective (or risk) factor. Given the heterogeneity in ASD-associated genetic events, it is likely that neither explanation is singularly sufficient and future work should continue investigating the impact of both psychiatric family burden and specific genetic events in the etiology of ASD.

The rate of depressive disorders was greater for paternal duplications than for maternal duplications, despite the fact that the rate of maternal depressive disorders was greater overall than paternal depressive disorders across genetic groups. These findings are particularly notable given the increased rates of maternal depression symptoms among the ASD population. Raising a child with ASD has been associated with increased parental stress and maternal depression compared to parents of children without ASD [39, 40]. Research suggests that mothers of children with ASD typically have greater parental strain than fathers associated with raising a child with ASD both in caregiving tasks and in expectations about family functioning. 79% of mothers report clinically significant depressive symptoms within one week of their child's ASD diagnosis and symptoms remain persistent for 37% of mothers one year later (Taylor and Warren, 2012). Approximately 29% of mothers report a delayed onset of depressive symptoms up to 16 months after the initial diagnosis. Factors such as child problem behaviors, ADHD symptoms [41], internalizing, externalizing, and behavior problems, access to services, financial barriers, family support, and parental adjustment [42] may interact in an additive or multiplicative way for mothers with children with ASD.

A relative strength of the current study is the ability to limit phenotypic and demographic confounders by closely matching participants in each group on a variety of domains. We focused only on individuals with clearly identified de novo ASD-associated events without additional inherited events. This study also expands the investigation of familial psychiatric history by including more family members (grandparents, aunts, and uncles), whereas most previous studies have focused on psychiatric history in only the parents [11, 13, 14, 18] and siblings [10, 16]. While previous studies have focused on parental psychiatric disorders in children with ASD compared to children without ASD [11, 13], this is the first study that compares the familial rate of psychiatric disorders between children with different types of genetic events associated with ASD.

Within this preliminary study, there are several limitations. First, we acknowledge that the population included in this study is limited by relatively small group sizes, particularly for the two de novo CNV groups (i.e., DUP,* n* = 62, and DEL,* n* = 74), and consists solely of children who participated in the Simons Simplex Collection (SSC). These children come from simplex families (i.e., only one known child or relative with ASD) and, subsequently, rates of familial psychiatric disorders may be lower than multiplex families. Considering the high functioning nature of the SSC cohort (i.e., large proportion of individuals with IQ > 70), future work should include increased sampling of children with ASD and intellectual disability. Second, although our structured interview was similar in scope to prior work (e.g., [17, 43]), our measurement consists of caregiver report of family mental health history information that could be influenced by possible reporting errors. Indeed, parents of a child with ASD report more psychiatric difficulties on self-report measures relative to clinical records [44], suggesting that the context of the interview (i.e., research versus clinical setting) may capture different aspects of the same outcome (i.e., subjective experiences versus clinical evaluation of functioning). Future work would benefit from a deeper understanding of the sensitivity of our measurement, as well as more detailed information such as the age of psychiatric disorder diagnosis. Lastly, although the groups were divided according to the specific type of structural mutation, it is possible that there remains a substantial amount of genetic heterogeneity within each group, which may be related to the extent by which familial factors contribute to ASD risk. Analysis of specific phenotypes tied to distinct genetic contributions and their relationship to family history may elucidate the relationship between ASD and other psychiatric disorders more clearly.

## 5. Conclusion

Our clarification of the differential association of family psychiatric history with distinct classes of genetic events in ASD provides a novel and informative step in elucidating the relationship between ASD and familial psychiatric burden. Our results support the proposal of the “second hit” hypothesis, such that elevated psychiatric burden (i.e., second hit) and the presence of a de novo copy number duplication (i.e., first hit) may generate an additive effect, subsequently resulting in the emergence of the ASD phenotype. In addition, our results suggest an elevated risk stemming from paternal family history of psychiatric burden, particularly related to depressive disorders. This is unusual in light of the fact that maternal rates of depressive disorders were greater across all genetic groups and highlights a critical avenue for future research. This work has important implications for diagnoses, pharmacological and psychological therapies, and strategies for investigating the etiology of neurodevelopmental disorders.

## Supplementary Material

Supplemental Table 1: Genetic characterization for de novo Deletion Group (DEL).
Supplemental Table 2: Genetic characterization for de novo Duplication Group (DUP).
Supplemental Table 3: Genetic characterization for de novo Likely-Gene Disrupting Mutations (LGDM).

## Figures and Tables

**Table 1:** Genetic group demographic characterization.

Number of subjects	DEL		DUP		LGDM		NON	
	*N*	%	*N*	%	*N*	%	*N*	%
*Total*	74		62		267		2111	
Female	15	20.3%	10	16.1%	48	18.0%	286	13.5%
Male	14	18.9%	52	83.9%	219	82.0%	1825	86.5%
*Race*								
African-American	1	1.4%	0	0.0%	13	4.9%	89	4.2%
Asian	3	4.1%	1	1.6%	16	6.0%	86	4.1%
Native (American or Hawaiian)	0	0.0%	0	0.0%	0	0.0%	5	0.2%
More than one race	5	6.8%	3	4.8%	25	9.4%	155	7.3%
White	60	81.1%	54	87.1%	200	74.9%	1647	78.0%
Not specified/other	5	6.8%	4	6.5%	13	4.9%	129	6.1%
*Household income*								
<$20,000	1	1.4%	3	4.8%	4	1.5%	63	3.0%
$21,000–$35,000	2	2.7%	3	4.8%	13	4.9%	107	5.1%
$36,000–$50,000	8	10.8%	5	8.1%	23	8.6%	173	8.2%
$51,000–$65,000	7	9.5%	8	12.9%	27	10.1%	222	10.5%
$66,000–$80,000	8	10.8%	7	11.3%	37	13.9%	272	12.9%
$81,000–$100,000	11	14.9%	12	19.4%	50	18.7%	346	16.4%
$101,000–$130,000	11	14.9%	7	11.3%	29	10.9%	314	14.9%
$131,000–$160,000	9	12.2%	6	9.7%	27	10.1%	198	9.4%
>$161,000	14	18.9%	8	12.9%	47	17.6%	324	15.3%
Not specified	3	4.1%	3	4.8%	10	3.7%	92	4.4%

Measure	DEL		DUP		LGDM		NON	
	Mean	SD	Mean	SD	Mean	SD	Mean	SD

Age	113.67	45.23	103.03	43.52	113.98	44.12	107.34	43.17
Verbal IQ	67.08	34.40	76.83	30.90	74.44	30.12	77.14	31.36
Nonverbal IQ	70.45	32.26	78.39	27.94	78.39	25.10	83.34	26.25
Internalizing problems	59.68	9.88	59.86	9.62	60.39	9.15	60.02	9.66
Externalizing problems	54.67	11.69	58.20	11.58	56.65	11.13	56.48	10.68

**Table 2:** Mixed model fixed effects results.

Effect	*F*	df	*p*
Kind of disorder	62.280	7, 40000	<.0001
Familial relation	10.700	1, 40000	.001
Kind of disorder × familial relation	5.170	7, 40000	<.0001
Genetic group	2.700	3, 40000	.044
Genetic group × kind of disorder	1.230	21, 40000	.209
Genetic group × familial relation	1.160	3, 40000	.323
Genetic group × kind of disorder × familial relation	0.630	21, 40000	.903

**Table 3:** Rate of psychiatric disorders group comparisons by familial relation.

	DEL	DUP	LGDM	NON	A priori comparisons
*Overall rates*	0.015	0.022	0.017	0.020	*NON < DEL*; *DEL < DUP*; LGDM < DUP
Anxiety disorders	0.028	0.033	0.023	0.029	
Bipolar disorders	0.014	0.025	0.016	0.017	
Depressive disorders	0.046	0.077	0.067	0.077	*DEL < DUP*; *DEL < LGDM*; *DEL < NON*; *LGDM < NON*
Feeding/eating disorders	0.007	0.005	0.000	0.004	
Neurodevelopmental disorders	0.007	0.014	0.018	0.016	
Obsessive compulsive disorders	0.004	0.011	0.006	0.007	
Schizophrenia/psychotic disorders	0.005	0.006	0.002	0.004	
Trauma/stress disorders	0.005	0.005	0.004	0.003	
*Maternal rates*	0.018	0.022	0.022	0.024	DEL < NON
Anxiety disorders	0.028	0.028	0.032	0.037	
Bipolar disorders	0.003	0.018	0.015	0.018	
Depressive disorders	0.069	0.087	0.081	0.096	*DEL < NON*; *LGDM < NON*
Feeding/eating disorders	0.015	0.004	0.001	0.005	
Neurodevelopmental disorders	0.006	0.014	0.025	0.020	DEL < LGDM
Obsessive compulsive disorders	0.008	0.015	0.010	0.008	
Schizophrenia/psychotic disorders	0.006	0.003	0.003	0.004	
Trauma/stress disorders	0.005	0.006	0.007	0.005	
*Paternal rates*	0.012	0.022	0.012	0.015	*DEL < DUP*; *LDGM < DUP*; NON < DUP
Anxiety disorders	0.029	0.038	0.014	0.020	*LGDM < DUP*; NON < DUP
Bipolar disorders	0.025	0.032	0.018	0.015	
Depressive disorders	0.022	0.067	0.052	0.057	*DEL < DUP*; *DEL < LGDM*; *DEL < NON*
Feeding/eating disorders	0.000	0.005	0.000	0.002	
Neurodevelopmental disorders	0.008	0.014	0.011	0.013	
Obsessive compulsive disorders	0.000	0.006	0.001	0.005	
Schizophrenia/psychotic disorders	0.005	0.009	0.002	0.005	
Trauma/stress disorders	0.005	0.004	0.001	0.002	
